# Efficacy and Safety of Vagus Nerve Stimulation on Upper Limb Motor Recovery After Stroke. A Systematic Review and Meta-Analysis

**DOI:** 10.3389/fneur.2022.889953

**Published:** 2022-07-01

**Authors:** Jorge A. Ramos-Castaneda, Carlos Federico Barreto-Cortes, Diego Losada-Floriano, Sandra Milena Sanabria-Barrera, Federico A. Silva-Sieger, Ronald G. Garcia

**Affiliations:** ^1^Fundación Cardiovascular de Colombia, Bucaramanga, Colombia; ^2^Research Group Innovación y Cuidado, Faculty of Nursing, Universidad Antonio Nariño, Neiva, Colombia; ^3^Department of Epidemiology, Universidad Surcolombiana, Neiva, Colombia; ^4^Department of Psychiatry, Massachusetts General Hospital, Harvard Medical School, Boston, MA, United States; ^5^School of Medicine, Universidad de Santander, Bucaramanga, Colombia

**Keywords:** vagus nerve, vagus nerve stimulation, transcutaneous vagus nerve stimulation, stroke, rehabilitation

## Abstract

**Background:**

Upper limb motor impairment is one of the main complications of stroke, affecting quality of life both for the patient and their family. The aim of this systematic review was to summarize the scientific evidence on the safety and efficacy of Vagus Nerve Stimulation (VNS) on upper limb motor recovery after stroke.

**Methods:**

A systematic review and meta-analysis of studies that have evaluated the efficacy or safety of VNS in stroke patients was performed. The primary outcome was upper limb motor recovery. A search of articles published on MEDLINE, CENTRAL, EBSCO and LILACS up to December 2021 was performed, and a meta-analysis was developed to calculate the overall effects.

**Results:**

Eight studies evaluating VNS effects on motor function in stroke patients were included, of which 4 used implanted and 4 transcutaneous VNS. It was demonstrated that VNS, together with physical rehabilitation, increased upper limb motor function on average 7.06 points (95%CI 4.96; 9.16) as assessed by the Fugl-Meyer scale. Likewise, this improvement was significantly greater when compared to a control intervention (mean difference 2.48, 95%CI 0.98; 3.98). No deaths or serious adverse events related to the intervention were reported. The most frequent adverse events were dysphonia, dysphagia, nausea, skin redness, dysgeusia and pain related to device implantation.

**Conclusion:**

VNS, together with physical rehabilitation, improves upper limb motor function in stroke patients. Additionally, VNS is a safe intervention.

## Introduction

Stroke is a neurological condition caused by vascular problems such as cerebral infarction and/or intracerebral or subarachnoid hemorrhage (1). In 2019, more than 12 million strokes occurred worldwide, making it one of the leading causes of morbidity. Stroke is considered the second leading cause of mortality overall and one of the leading causes of disability worldwide, ranking first in people over 50 years of age (2). In the United States, stroke occurs in more than 7 million people, with a prevalence of 2.5 % (3).

Motor impairment occurs in 85% of patients with stroke, and it is considered one of the main problems resulting from this condition (4). Motor affectation in these subjects is characterized by a decreased capacity and strength of muscles, mainly of the upper extremities, diminishing the quality of life of both the patients and their families (5). Recovery of motor function occurs spontaneously during the 1st months after stroke (6) as a result of brain plasticity processes in the sensory and motor systems (7), however, 50 to 75% of these patients persist with significant motor sequelae limiting daily activities (8).

Efforts have been made to develop therapies that can improve motor impairment in stroke patients (9). Among these therapies are: constraint-induced movement (10), mirror therapy (11), and resistance training (12), however, these interventions have a low level of adherence (13) and the evidence supporting their effects is still weak (10–12). Recently, Vagus Nerve Stimulation (VNS) has been proposed as an intervention that could have beneficial effects in the recovery of motor function in these patients, since it contributes to the generation of adaptive neuroplasticity and the activation of neuromodulators that reduce brain inflammation (14, 15).

VNS consists in the activation of the vagus nerve using electrical current, either through the use of implants or extracorporeal electrodes. As the vagus nerve is composed mainly of afferent fibers, it allows the modulation of different brain structures receiving vagal afferent information, such as the nucleus of the solitary tract, locus coeruleus, raphe nuclei and the hypothalamus (16). In experimental animal stroke models, VNS has been shown to reduce infarct volume and improve neurological outcomes (17, 18). It has been proposed that one the mechanisms mediating these neuroprotective effects of VNS in acute cerebral ischemia is the modulation of the cholinergic anti-inflammatory pathway, and more specifically the α7 nicotinic acetylcholine receptor (α7nAChR) (19), a neurotransmitter gated ion channel expressed widely in the brain and on immune cells (20, 21). Activation of these receptors by the vagus nerve leads to a reduction in the release of pro-inflammatory cytokines (21), with beneficial effects on the reduction of infarct size and cerebral edema on experimental models of stroke (19).

In addition, it has been shown that VNS paired with motor training of the extremities, may upregulate cortical plasticity mechanisms that result in motor function recovery after a stroke (22). Following brain injuries affecting the motor or sensory cortices, nearby cortical regions partially regenerate to provide some of the lost functionality (23, 24). The size of the regenerated motor or sensory representation in surrounding cortical areas correlates with functional recovery, however the result gain in functionality is only a fraction of the observed pre-injury levels (24, 25). Previous studies have demonstrated that repeatedly pairing VNS with specific movements results in increased representation of these movements in the primary motor cortex (22). Further animal experiments have provided evidence that the administration of VNS paired with repeated movements of affected limbs after motor cortex damage is associated with a significant recovery of forelimb function that is superior to that observed with physical training alone (26, 27). These potentiating effects of VNS on cortical reorganization mechanisms may be related with the activation of nuclei such as locus coeruleus, raphe nuclei and nucleus basalis. These nuclei generate an increase in neuromodulators important in neuroplasticity, such as noradrenaline, serotonin, brain-derived neurotrophic factor and acetylcholine (28, 29). When these neurotransmitters are simultaneously released during neural activity related with motor rehabilitation, synaptic plasticity is promoted in motor-specific circuits (30). Thus, VNS paired with motor rehabilitation can cause an increased specific reorganization of the motor cortex, resulting in an enhanced motor recovery after cerebral ischemia (27).

VNS has mainly been administered by using implanted electrodes, but more recently, a non-invasive technique, known as transcutaneous VNS (cervical or auricular) has been proposed (31). VNS has traditionally required the implantation of an electrical pulse generator at the left subclavicular level, which is connected to electrodes in the left cervical branch of the vagus nerve (32). Its insertion is performed by a surgical procedure, which presents a higher risk of adverse events (33), the most frequent being dysphonia during stimulation, due to its proximity to the laryngeal nerve (34). On the other hand, transcutaneous VNS works through the placement of non-invasive electrodes on the neck or auricle for stimulation of the cervical or auricular branch of the vagus nerve, respectively (32). Transcutaneous VNS has a lower risk of adverse events, is reversible and easy to implement (32). In addition, experimental evidence suggests that the effects of transcutaneous VNS on brain function are comparable to those obtained with VNS (33). Diverse studies using electrical stimulation of the auricular branch of the vagus nerve in experimental models have shown a significant effect of this technique on the reduction of brain infarct volume (35–37). The magnitude of reduction in infarct size has been similar to the one reported for implanted VNS (18). In addition to these effects, transcutaneous VNS has shown to regulate other mechanisms that can promote recovery of neurological function after ischemic stroke (38, 39). These include upregulation of angiogenesis, which can improve perfusion of the tissue surrounding the injury promoting recovery (40), regulation of blood brain barrier permeability, which could improve cerebral edema after stroke (41), and inhibition of neuroinflammation resulting in neuroprotective effects against ischemic cerebral injuries (37). No animal studies have evaluated the effects of transcutaneous VNS paired with rehabilitation on the recovery of motor function after brain ischemic injury. However, multiple studies have shown beneficial effects of this technique on upregulation of mechanisms involved in neuroplasticity, such as upregulation of brain-derived neurotrophic factor (42). Transcutaneous VNS has also shown to improve axon regeneration and re-organization in experimental models of cerebral ischemia (37), suggesting that this technique may have similar effects to VNS on the mechanisms underlying its beneficial effects on motor recovery after a stroke.

There have been multiple clinical studies that have evaluated the safety and efficacy of implanted and transcutaneous VNS in the recovery of motor function after stroke (34, 43–49), and recently, meta-analyses have suggested that VNS has a positive effect on upper limb function in stroke patients (50–52), however these reviews did not evaluate the effect vagus nerve stimulation according to time since stroke. The aim of this systematic review is to summarize and analyze the scientific evidence of the safety and efficacy of both implanted and transcutaneous VNS for the management of upper limb motor impairment after stroke.

## Methods

### Search Strategy

The search was performed using the following databases: MEDLINE, CENTRAL, EBSCO and LILACS, without date restriction and was focused on studies conducted in humans. A combination of MeSH terms was used for the search, which were: ((Vagus Nerve Stimulation) OR (Vagus Nerve)) AND (Stroke). The search was conducted in December 2021 and was restricted to articles published in English or Spanish.

### Selection Criteria

Studies of patients with acute or chronic stage stroke, where VNS was the intervention, compared to usual care or placebo stimulation, were included. The main outcome was the efficacy of VNS on upper limb motor recovery. Information on mild, moderate and severe adverse events of VNS was also collected to assess safety aspects. Clinical trials were included. Editorials, protocols, letters to the editor, commentaries, and case reports were excluded. Studies that only evaluated neuroplasticity mechanisms, neuromodulator production, cytokine inhibition, or brain infarct volume were also excluded. In order to include all relevant research, we reviewed the references of the included studies and also published abstracts from scientific conferences. In addition, we searched the clinicaltrial.gov website to identify clinical trials of VNS in stroke patients. The first author (JAR) independently reviewed the titles and abstracts for an initial assessment of eligibility criteria. Once the titles and abstracts were reviewed, JAR and DL reviewed the full-text articles to evaluate the inclusion of studies in the analysis. Discrepancies and doubts on the inclusion of articles were resolved by a third investigator (RG).

### Data Extraction

Information from the articles was extracted by two reviewers (JAR, DL), using an established format containing the following variables: lead author, year of publication, outcome assessed, type of study, population, intervention assessed, comparison group, results in terms of primary and secondary outcomes, adverse event reporting, and stimulation parameters.

### Assessment of Methodological Quality

We assessed the risk of bias of the included clinical trials using the Cochrane Collaboration's domain-based scale, which evaluates allocation concealment, randomization, blinding of participants and investigators, blinding in outcome assessment, selective outcome reporting, and incomplete outcome data.

### Synthesis of Information

Qualitative analysis of each of the articles was performed, taking into account the characteristics of the studies, population, intervention, control group, outcomes and adverse event reporting.

### Quantitative Analysis

An initial meta-analysis of 6 clinical trials was performed (three implanted and three transcutaneous VNS studies), where the mean difference in upper limb motor recovery between the active and control interventions was assessed. A second meta-analysis evaluated the average increase in motor recovery from baseline and included six clinical trials and one intervention study that had no comparison group, for a total of seven studies (three evaluating implanted VNS and four evaluating transcutaneous VNS). In each meta-analysis, the mean with its 95 % confidence interval was calculated. Care was taken not to duplicate data from clinical trials with more than one publication. A subgroup analysis was performed to determine the difference in the effects according to the VNS technique (implanted vs. transcutaneous) and the mean time since the stroke (more than 3 years versus <3 years). A random-effects model was used for the meta-analysis and heterogeneity was assessed using the I^2^ statistic, where I^2^ >60% was considered as significant heterogeneity. All analyses were performed in the RStudio program using the meta library.

## Results

**Figure 1:**
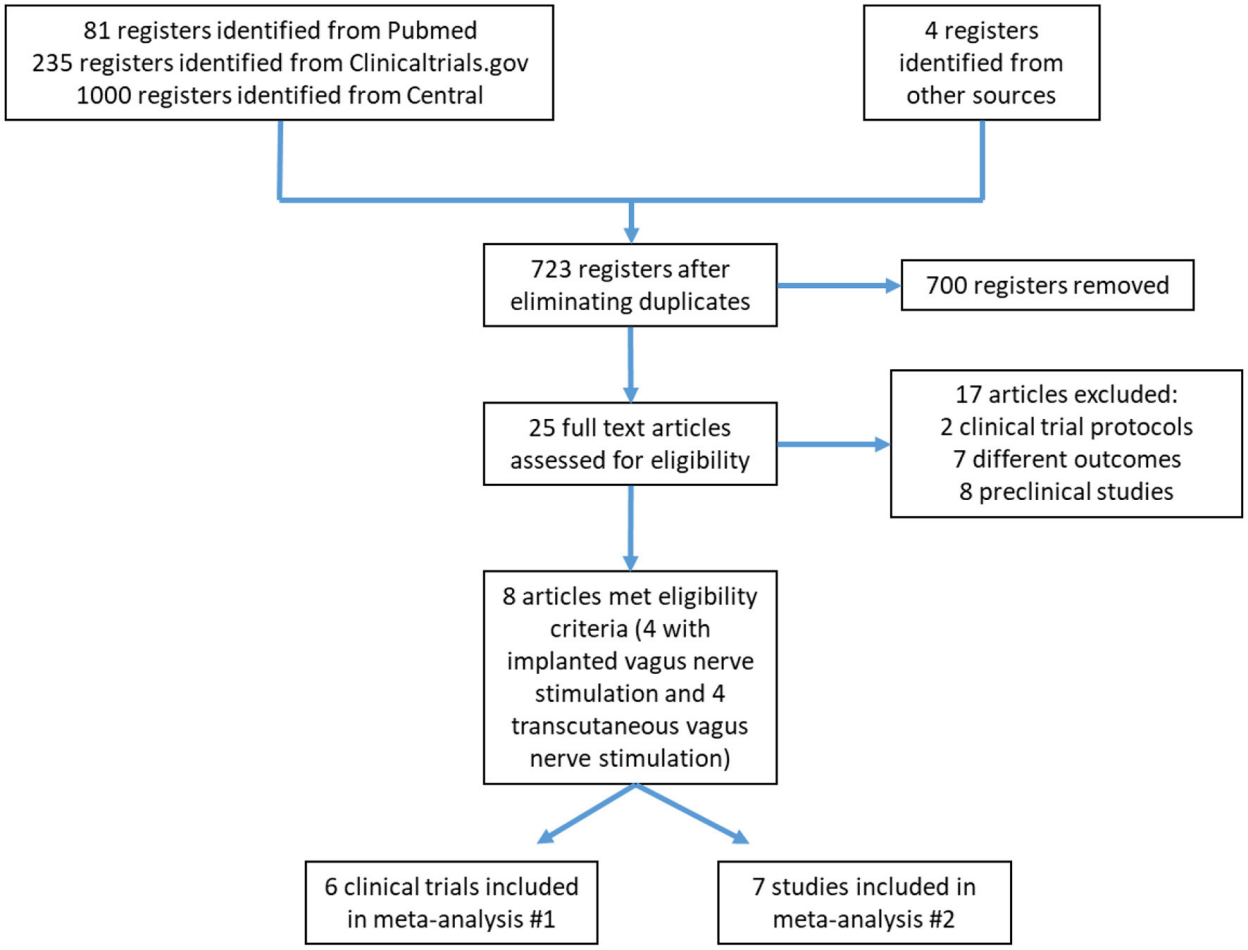
Flow diagram of study searching and selection process.

The database search yielded 1,316 records; after eliminating duplicates, 723 were selected for title and abstract review. Of these, 700 were excluded mainly because of study design and the lack of stroke as a studied event. In total, 25 research articles were reviewed in full. From these, eight articles met the eligibility criteria for the systematic review (Figure 1). The main reasons for exclusion were the evaluation of outcomes other than those stated in the selection criteria of this review (e.g., evaluation of physiological mechanisms of neuroplasticity or impact on cerebral infarct size).

### Study Characteristics

Eight studies evaluating VNS were included, all of them published in English between 2016 and 2021, of which four used implanted and four transcutaneous VNS [cervical (*n* = 0), auricular stimulation (*n* = 4)]. The implanted VNS studies analyzed the efficacy of stimulation plus physical rehabilitation compared with patients who received physical rehabilitation therapies plus placebo stimulation or physical therapy alone (34, 47–49). The implanted VNS protocol in all evaluated studies had a duration of 18 sessions distributed over 6 weeks, where stimulation was administered in conjunction with rehabilitation training and used the following parameters: an amplitude of 0.8 mA, a pulse duration of 0.1 ms, a frequency of 30 Hz, and a duration of 0.5 seconds; with stimuli administered during each movement repetition. In general, all patients who received the intervention had a significant improvement in motor function, as assessed by the Fugl-Meyer scale (Table 1). This improvement in motor function persisted significantly up to 90 days after the end of the intervention in two studies (34, 49) (Table 1).

**Table 1:** Characteristics of the studies included in the systematic review.

**Author (year)**	**Outcome**	**Population**	**Intervention group**	**Control group**	**Results**
Dawson et al. (47) (NCT01669161)	Upper limb motor function	Twenty patients with a history of unilateral supratentorial ischemic stroke that occurred at least 6 months before inclusion.	Nine patients with implanted VNS on the left vagus nerve (0.5 s of charged balanced pulses with 0.8 mA amplitude, 100 μs pulse width, 30-Hz frequency, delivered during each movement repetition) + rehabilitation therapy (6-week course of 2-h therapy sessions, 3x week, and at least 300 to 400 movements per session).	Eleven patients with rehabilitation therapy only (6-week course of 2-h therapy sessions, 3x week, at least 300 to 400 movements per session). This group did not have an implanted device.	The mean change in the Fugl-Meyer Assessment-Upper Extremity (FMA-UE) score in the VNS group was 8.7 (SD 5.8) vs. 3.0 (SD 6.1) in the control group (between group difference = 5.7, 95% CI−0.4; 11.8, *p* = 0.064)
Kimberley et al. (49) (NCT02243020)	Upper limb motor function	Seventeen patients with a history of unilateral supratentorial ischemic stroke that occurred between 4 months to 5 years before randomization	Eight patients with implanted VNS on the left vagus nerve (0.5 s of charged balanced pulses with 0.8 mA amplitude, 100 μs pulse width, 30-Hz frequency, delivered during each movement repetition) + rehabilitation therapy (6-week course of 2-h therapy sessions, 3x week, and 300 to 500 movement repetitions per session). After 6 weeks of in-clinic therapy, participants began daily therapist-prescribed home exercises. For the first 30 days of at-home therapy, participants received 0 maVNS and active VNS thereafter.	Nine patients with sham stimulation (0 mA) + rehabilitation therapy (6-week course of 2-h therapy sessions, 3x week, and 300 to 500 movements per session). After 6 weeks of in-clinic therapy, participants began daily therapist-prescribed home exercises.	**Day 1 after therapy:** The mean change in FMA-UE score in the VNS group was 7.6 vs. 5.3 in the sham group (between group difference = 2.3, 95% CI−1.8; 6.4, *p* = 0.20). **Day 90 after therapy:** The mean change in FMA-UE score in the VNS group was 9.5 vs 3.8 in the sham group (between group difference = 5.7, 95% CI−1.4; 11.5, *p* = 0.055). The FMA-UE response rate at day 90 (≥6-point change from baseline) in the VNS group was significantly higher (88.0%) compared with the control group (33.0%) (*p* = 0.03)
Dawson et al. (48) (NCT02243020)	Upper limb motor function	Seventeen patients with a history of unilateral supratentorial ischemic stroke that occurred between 4 months to 5 years before randomization	Eight patients with implanted VNS initially underwent 6 weeks of in clinic rehabilitation therapy + active VNS followed by home exercises paired with self-administered active VNS.	Nine patients with implanted VNS initially underwent 6 weeks of in clinic rehabilitation therapy + sham VNS followed by home exercises with control VNS through day 90. Subjects in this group then crossed over and received 6-weeks of in-clinic rehabilitation paired with active VNS and continue a home exercise program paired with self-administered active VNS	**1-year follow-up of VNS paired with rehabilitation for all participants:** The FMA-UE score increased by 9.2 points (95% CI = 4.7; 13.7; *P* = 0.001). 73% demonstrated a clinically meaningful improvement (≥6 points) in FMA-UE
Dawson (2021) (34) (NCT03131960)	Upper limb motor function	Hundred and eight patients with history of unilateral supratentorial ischemic stroke that occurred between 9 months and 10 years before enrolment.	Fifty-three with implanted VNS on the left vagus nerve (0.5 s of charged balanced pulses with 0.8 mA amplitude, 100 μs pulse width, 30-Hz frequency, delivered during each movement repetition) + rehabilitation therapy (6-week course of 2-h therapy sessions, 3x week, and > 300 movement repetitions per session). After 6 weeks of in-clinic therapy, participants began daily therapist-prescribed home exercises. For the first 30 days of at-home therapy, participants received 0 maVNS and active VNS thereafter.	Fifty-five patients with sham stimulation (0 mA) + rehabilitation therapy (6-week course of 2-h therapy sessions, 3x week, and >300 movement repetitions per session). After 6 weeks of in-clinic therapy, participants began daily therapist-prescribed home exercises.	**Day 1 after therapy:** The FMA-UE score was significantly increased in the VNS group compared with the control group (5.0 [SD 4.4] vs. 2.4 [SD 3.8]); between group difference = 2.6, 95%CI 1.0; 4.2, (*p* = 0.0014). **Day 90 after therapy:** The FMA-UE score was significantly increased in the VNS group compared with the control group (5.8 [SD 6.0] vs. 2.8 [SD 5.2]); between group difference = 3.0, 95%CI 0.8; 5.1, (*p* = 0.0077). The FMA-UE response rate (≥6-point change from baseline) in the VNS group was significantly higher (47.0%) compared with the control group (24.0%) (between group difference 24.0%, 95%CI 6; 41, *p* = 0.0098).
Capone et al. (46)	Upper limb motor function	Fourteen patients with either ischemic or hemorrhagic stroke that occurred at least 1 year before inclusion.	Seven patients with transcutaneous auricular VNS (location = left external acoustic meatus, frequency = 20 Hz, pulse width = 0.3 ms, duration = 20 s, intensity = level between the detection and pain thresholds) repeated every 5 min for 60 min + robot-assisted therapy (three sessions of 320 assisted movements per day) Immediately after the stimulation. The intervention was delivered daily for 10 consecutive working days	Seven patients with sham stimulation (location = left ear lobe, frequency = 20 Hz, pulse duration = 0.3 ms, duration = 20 s, intensity = level between the detection and pain thresholds) repeated every 5 min for 60 min + robot-assisted therapy (three sessions of 320 assisted movements per day) Immediately after the stimulation. The intervention was delivered daily for 10 consecutive working days.	The FMA-UE score was significantly increased in the VNS group compared with the control group (5.4 vs 2.8; Mann– Whitney U = 5 00, *p* = 0.048)
Redgrave et al. (45) (NCT03170791)	Upper limb motor function	13 patients with an anterior circulation ischemic stroke at least 3 months before enrolment	13 patients with transcutaneous auricular VNS (location = left cymba concha, frequency = 25 Hz, pulse width = 0.1 ms, intensity = maximum tolerable level) delivered during each movement repetition + rehabilitation therapy (6-week course of 1-h therapy sessions, 3x week consisting of upper limb repetitive task practice: 30–50 repetitions of 7–10 arm movements)	No control group	The mean (SD) improvement in FMA-UE was 17.1 (SD 7.8). Ten patients (83%) achieved a clinically relevant increase of >10 points with an overall effect size of 0.68
Wu (57) (registration no. ChiCTR1800019635)	Upper limb motor function	Twenty two patients with a history of ischemic stroke that occurred between 0.5 and 3 months before enrollment	Ten patients with transcutaneous auricular VNS (location = left cymba concha, frequency = 20 Hz, pulse width = 0.3 ms, intensity = maximum tolerable level, lasting 30 seconds each time, stimulating once every 5 min) performed for 30 min + rehabilitation therapy (30 min, performed after the end the stimulation) per day for 15 consecutive days	Eleven patients with sham stimulation (electrodes were fixed to the cymba conchae of the left ear without electrical stimulation) performed for 30 min + rehabilitation therapy (30 min, performed after the end the stimulation) per day for 15 consecutive days	**Day 1 after therapy:** The FMA-UE score was significantly increased in the VNS group compared with the control group (6.9 [SD 1.85] vs 3.18 [SD 1.17]); between group difference = 3.72, 95%CI 2.32; 5.12, *p* < 0.001). **Week 4 after therapy:** The FMA-UE score was significantly increased in the VNS group compared with the control group (7.70 [SD 1.49] vs. 3.36 [SD 1.75]); between group, *p* < 0.001)
Chang et al. (44)(NCT03592745)	Upper limb motor function	Thirty-four patients with unilateral supratentorial stroke and chronic (>6 months) upper limb hemiparesis	Seventeen patients with transcutaneous auricular VNS (location = left cymba concha, frequency = 30 Hz, pulse width = 0.3 ms, intensity = maximum tolerable level) ~ 250 stimulated movements per session + shoulder/elbow robotic therapy (total of 1,024 flexion, extension, and rotational movements of the elbow and shoulder joints) 3 days per week for 3 weeks (9 sessions)	Seventeen patients with sham stimulation (location = left cymba concha, intensity = 0 ma) + shoulder/elbow robotic therapy (total of 1,024 flexion, extension, and rotational movements of the elbow and shoulder joints) 3 days per week for 3 weeks (9 sessions)	**At discharge:** The FMA-UE score was increased in the VNS group compared with the control group (3.10 [SEM 0.57] vs. 2.86 [SEM 0.50]). **Follow up (3 months after intervention):** The FMA-UE score was increased in the VNS group compared with the control group (2.79 [SEM 0.84] vs. 3.22 [SEM 1.0])

*SEM, Standard error of the mean*.

Regarding transcutaneous VNS, all studies used auricular stimulation. The study by Redgrave et al. (45) included 13 patients that had an anterior circulation ischemic stroke at least 3 months previously and had residual upper limb dysfunction. These subjects underwent 18 x 1-hour sessions over 6 weeks in which they received stimulation on the cymba conchae of the left ear concurrently with upper limb repetitive task practice (30–50 repetitions of 7–10 arm movements). Subjects received transcutaneous VNS with a frequency of 25 Hz, a pulse width of 0.1 ms, at maximum tolerated intensity (median intensity = 1.4 mA) during each movement repetition. This study found that transcutaneous VNS improved mean motor mobility at visit 18 (upper limb Fugl-Meyer mean increase = 17.1, SD 7.8), and that 10 patients (83 %) achieved a clinically relevant increase of >10 points on the Fugl-Meyer scale (45).

The study by Capone et al. (46) was a controlled clinical trial with a sample of 14 patients. Patients were randomized to robot-assisted physical therapy sessions associated with active transcutaneous auricular VNS or sham stimulation during 10 consecutive working days. Stimulation consisted of pulse trains lasting 20 s, with a pulse width of 0.3 ms and a frequency of 20 Hz, repeated every 5 min for 60 min. Patients in the transcutaneous auricular VNS group received the stimulation with electrodes placed in the left external acoustic meatus at the inner side of the tragus, whereas for those in the control group, electrodes were attached to the left ear lobe. The intensity of the stimulation was adjusted to a level between the detection and pain thresholds. Robotic-assisted therapy was delivered immediately after the end of real or sham transcutaneous VNS. In this study, the active intervention was found to improve upper extremity motor mobility (Fugl-Meyer scores) after 2 weeks of treatment as compared to the sham group (Mann-Whitney U = 5.00, *p* = 0.048) (34) (Table 1). Additionally, no adverse events were reported, and patients reported comfort and convenience during the intervention.

Wu et al. (43) evaluated the efficacy of transcutaneous auricular VNS in 10 patients with a history of ischemic stroke that occurred between 0.5 and 3 months compared with 11 patients that received sham stimulation. The active transcutaneous auricular VNS was delivered with electrodes fitted to the left cymba concha and consisted of pulse trains lasting 30 s, with a pulse width of 0.3 ms and a frequency of 20 Hz, repeated every 5 minutes for 30 minutes. The control group received sham stimulation (location= left cymba concha, intensity = 0 mA). Both groups received rehabilitation therapy performed after the end of the stimulation. In the study, the intervention group was found to significantly increase on FMA-UE score compared with the control group (6.9 [SD 1.85] vs. 3.18 [SD 1.17]); between group difference= 3.72, 95%CI 2.32; 5.12, *p* < 0.001) (Table 1).

Chang et al. (44) is the most recent study that evaluated the efficacy of the transcutaneous auricular VNS on the upper limb motor function. In this clinical trial, the authors included 34 patients with unilateral supratentorial chronic (>6 months) stroke. The intervention consisted of transcutaneous auricular VNS (location = left cymba concha, frequency = 30 Hz, pulse width = 0.3 ms, intensity = maximum tolerable level) with robotic therapy 3 days per week, for 3 weeks (nine sessions). The control group received sham stimulation (location = left cymba concha, intensity = 0 ma) with robotic therapy 3 days per week, for 3 weeks (nine sessions). The study found that at discharge, the FMA-UE score was increased in the intervention group compared with the control group (3.10 [SEM 0.57] vs. 2.86 [SEM 0.50]) (Table 1).

**Figure 2:**
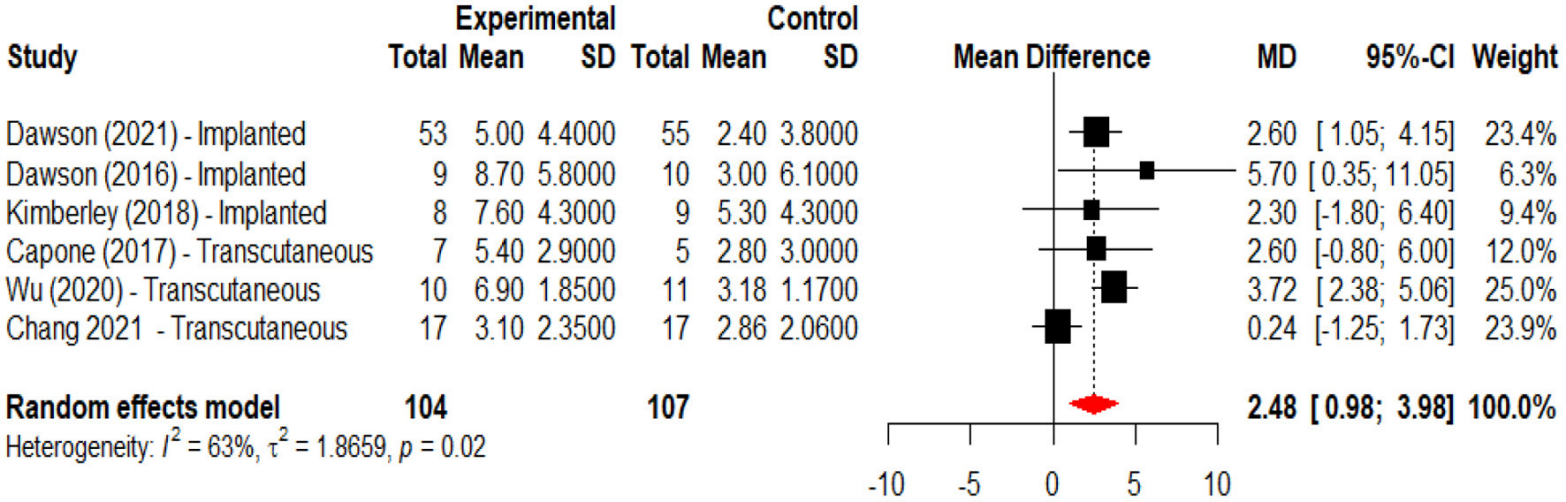
Forest plot for the meta-analysis of vagus nerve stimulation effects on upper limb motor function (FMA-UE score increase) when compared to a control intervention. Dawson et al. (34), Dawson et al. (47), and Kimberley et al. (47) used implanted stimulation, Capone et al. (46), Wu et al. (43), and Chang et al. (44) used transcutaneous stimulation.

**Figure 3:**
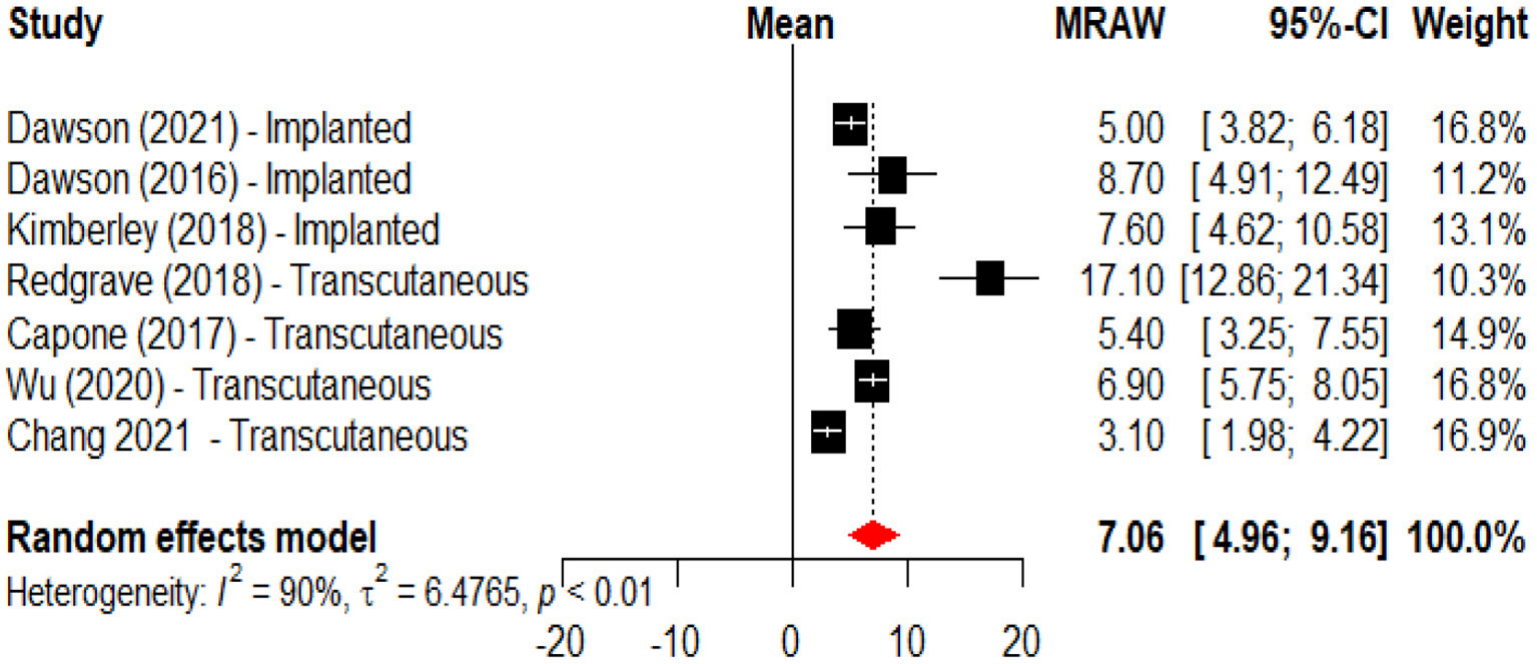
Forest plot for the meta-analysis of vagus nerve stimulation effects on upper limb motor function (FMA-UE score increase) when compared to baseline. Dawson et al. (34), Dawson et al. (47), and Kimberley et al. (47) used implanted VNS, Capone et al. (46), Redgrave et al. (45), Wu et al. (43), and Chang et al. (44) used transcutaneous stimulation.

**Figure 4:**
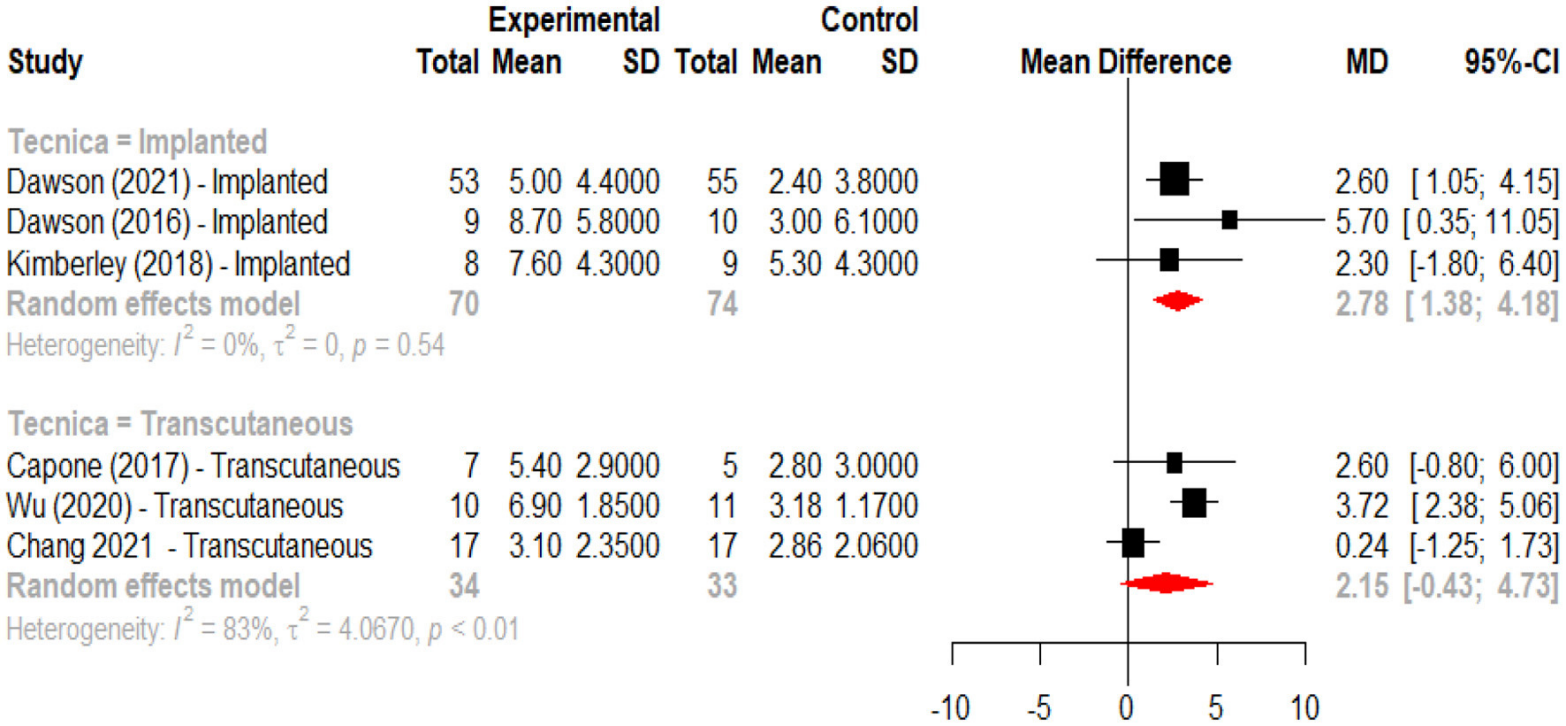
Forest plot for the meta-analysis of vagus nerve stimulation effects on upper limb motor function (FMA-UE score increase) when compared to a control intervention according to intervention modality (implanted vs. transcutaneous).

**Figure 5:**
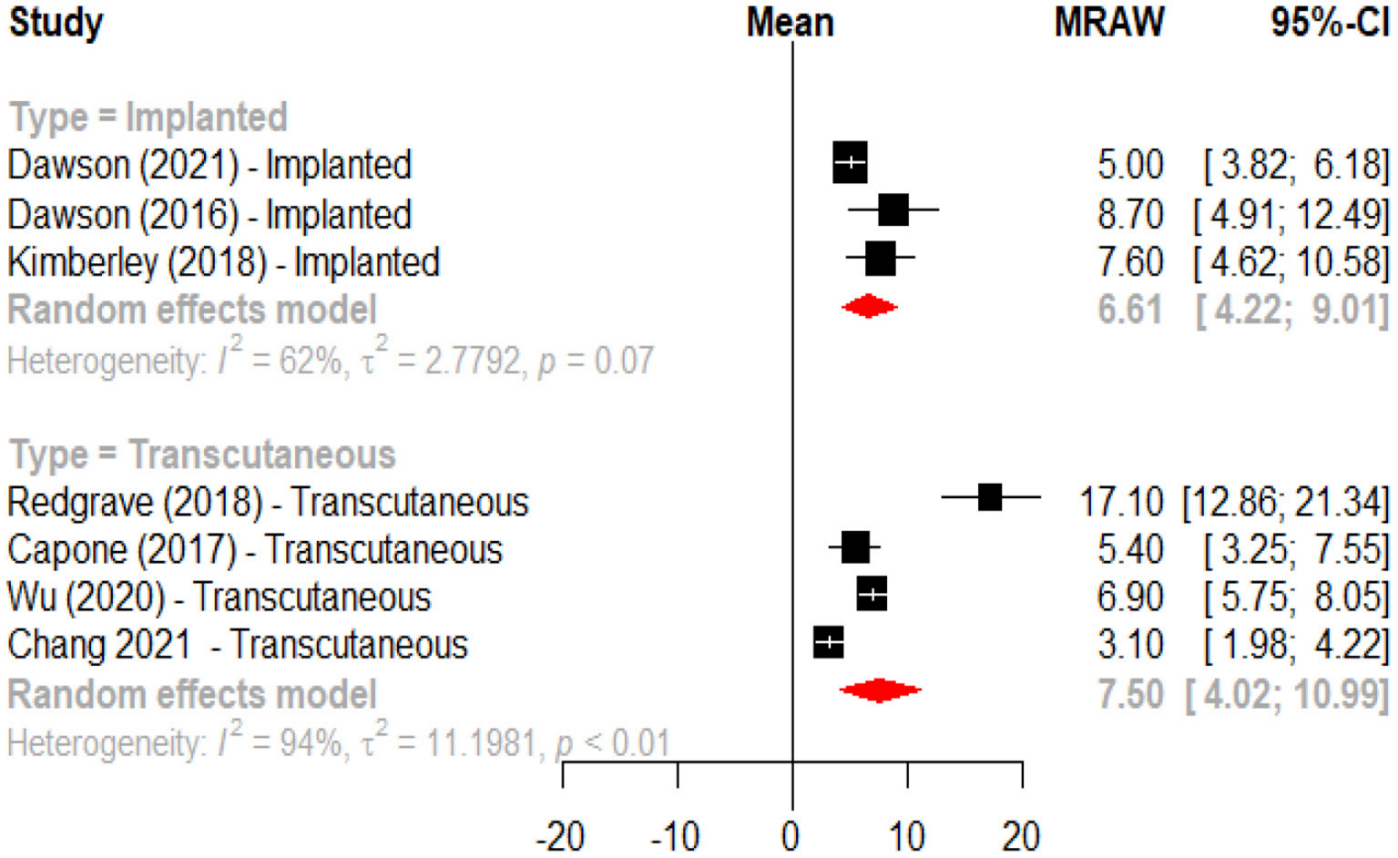
Forest plot for the meta-analysis of vagus nerve stimulation effects on upper limb motor function (FMA-UE score increase) when compared to baseline according to intervention modality (implanted vs. transcutaneous).

**Figure 6:**
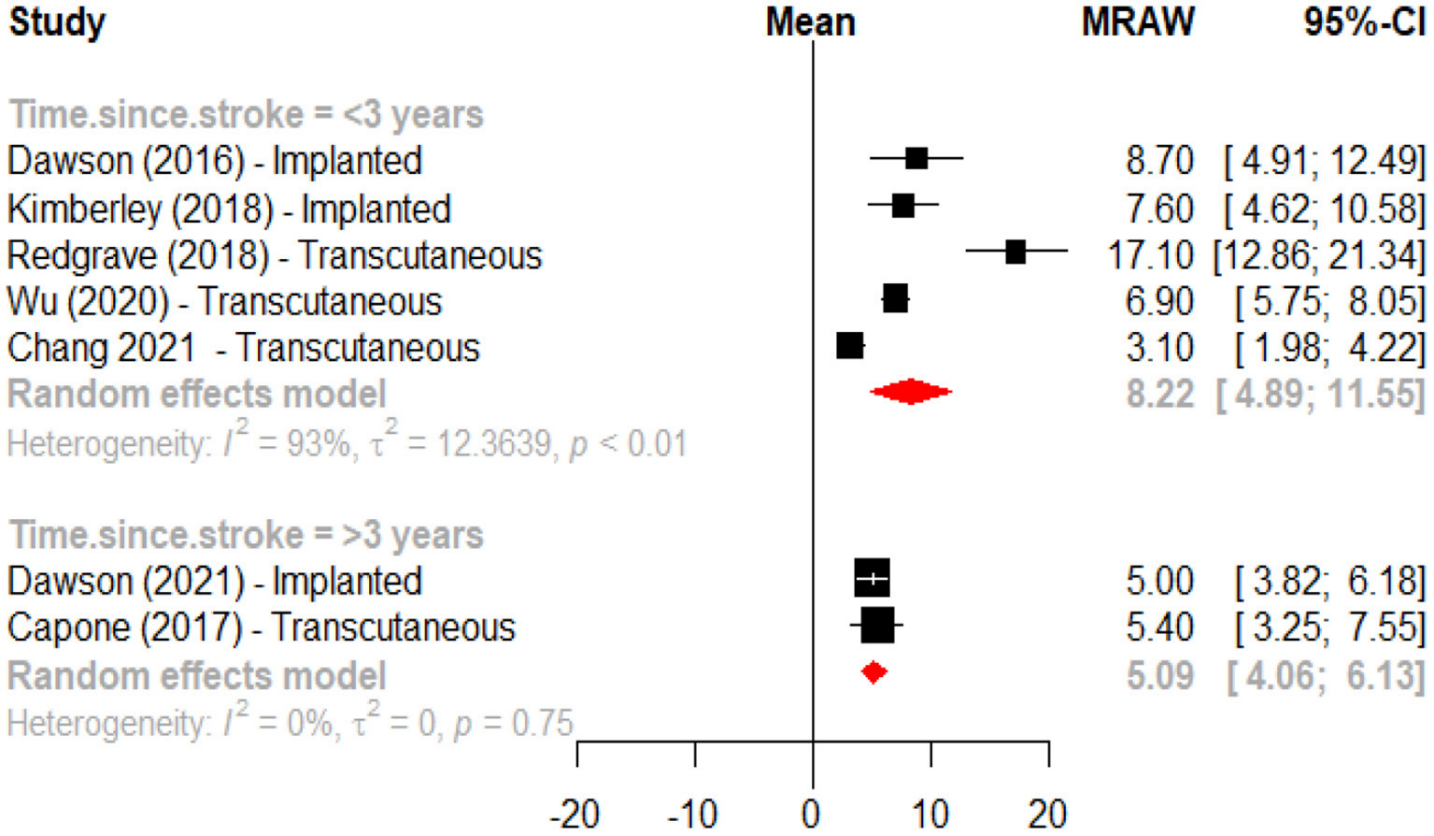
Forest plot for the meta-analysis of vagus nerve stimulation effects on upper limb motor function (FMA-UE score increase) when compared to baseline according to time since stroke (<3 years vs. ≥3 years).

An initial meta-analysis was performed to evaluate the effects of active VNS vs. a control intervention on motor recovery after stroke. From the eight studies one was excluded because it did not have a control group (45), and one more (48) because reported data from clinical trials with a publication already included in the analysis. This meta-analysis revealed that motor recovery, as measured by change in the Fugl-Meyer assessment score, was significantly greater in the active group when compared to those subjects receiving the control intervention (mean difference 2.48, 95%CI 0.98; 3.98) (Figure 2). In a second meta-analysis, including the study that did not have a control group (45), it was observed that the intervention increased upper limb motor function (Fugl-Meyer scores) by an average of 7.06 (95%CI 4.96; 9.16) points when compared to baseline (Figure 3). An analysis by subgroups in meta-analysis #1 did not show clear differences (Figure 4). The analysis by subgroups in meta-analysis #2 showed that studies where transcutaneous VNS (Figure 5) was used or included participants with a lower average time since stroke (<3 years) (Figure 6) were associated with greater effects in motor recovery.

### Safety

No intervention-related deaths or serious adverse effects (AEs) were reported. The most frequent moderate AE associated with implanted VNS was left vocal cord paralysis associated with or without dysphonia (11.11 % in Dawson et al. and 5.88 % in Kimberley et al.) (47, 49); The most frequent moderate AE associated with the device implantation procedure, present in one patient, was surgical site infection requiring intravenous antibiotic treatment (49). The most frequent mild AEs related to stimulation therapy were, in order: dysphonia, dysphagia, nausea and dysgeusia. None of the mild AEs required changes in therapy protocol and all of them self-resolved during the follow-up period (34, 47, 49). Four studies reported AEs in transcutaneous VNS, observing fatigue, dizziness, ear pain, skin redness and tiredness (43, 45, 53). In the study by Capone et al. (transcutaneous VNS) there were no adverse events and patients did not report discomfort from the procedure (46). One study that monitored heart rate and blood pressure levels showed no clinically significant change throughout the treatment sessions (43).

### Risk of Bias Assessment

**Figure 7:**
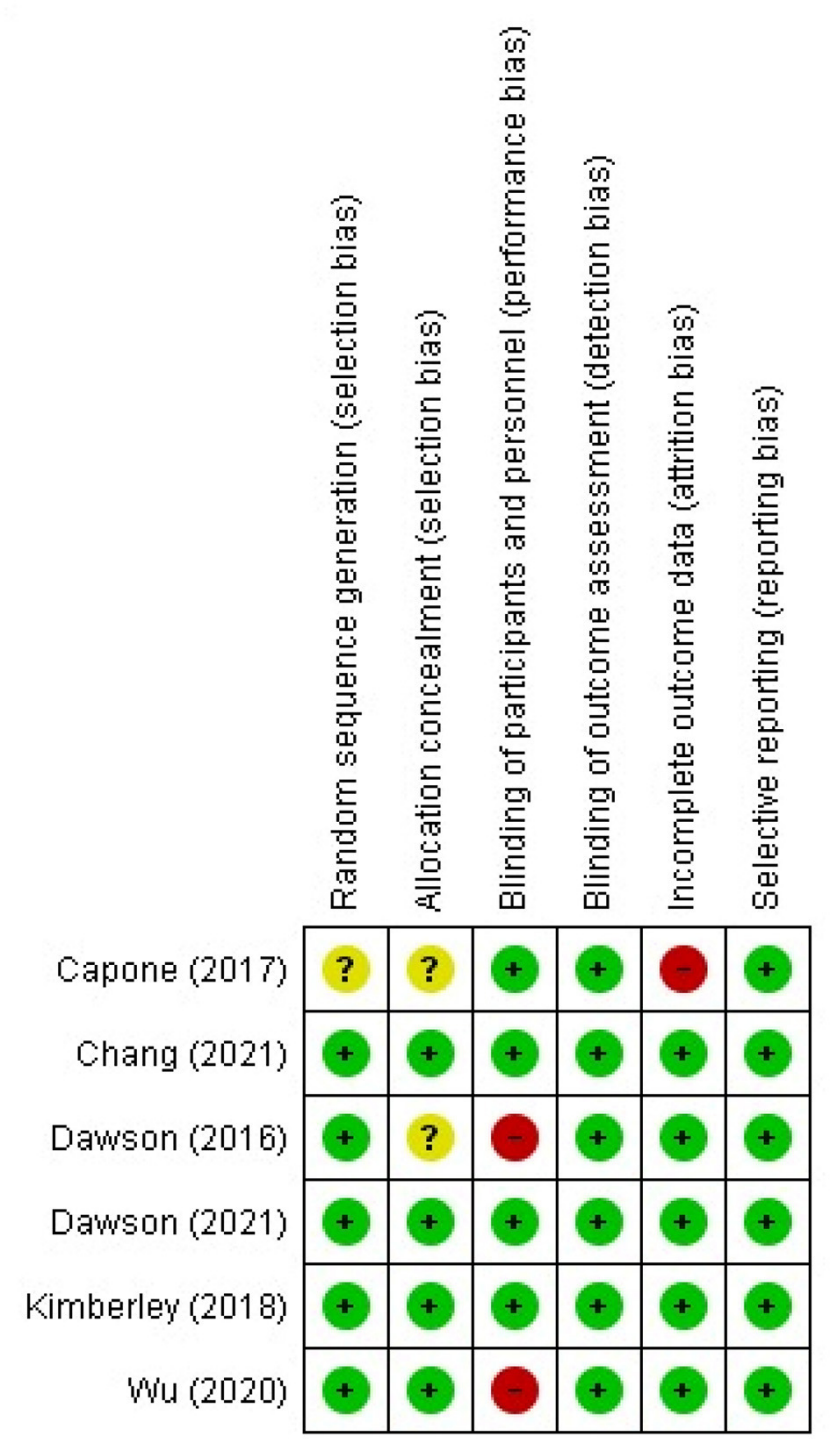
Risk of bias of the clinical trials included in the meta-analysis.

The risk of bias assessment was applied to the six clinical trials (Dawson et al., Dawson et al., Kimberley et al., Capone, et al., Wu et al., Chang et al.) (34, 43, 44, 46, 47, 49) upon which the initial meta-analysis for the assessment of VNS efficacy for motor rehabilitation was based. All studies had low risk of bias in the selective reporting domain. The performance bias domain had the highest risk of bias with two studies (Dawson et al., Wu et al.) (43, 47) (Figure 7). In the clinical trial reported by Capone et al. (46), the risk of bias in the domains of randomization, and allocation concealment was unclear, and the risk of attrition bias was high (Figure 7).

## Discussion

This systematic review and meta-analysis demonstrate that VNS is an effective therapy for upper limb motor recovery in stroke patients. Factors such as the VNS technique used and the time of intervention since the event seem to have an influence on the results obtained, with greater benefits if the stimulation is performed non-invasively and prior to 3 years after the event. However, the studies performed so far with transcutaneous stimulation have included a limited number of patients, therefore more evidence is needed before a definitive conclusion can be reached in this regard. The performed studies have shown a low rate of adverse events, so it can be concluded that VNS is a safe procedure for the management of this pathology. The incidence and severity of adverse events depend on whether the stimulation is performed with an implanted device or with a non-invasive technique, since the former has a higher risk of moderate adverse events such as vocal cord paralysis and surgical site infection associated with the implantation procedure, whereas for the transcutaneous technique the adverse events reported were all mild (e.g., fatigue, dizziness, ear pain and tiredness).

Previous clinical studies have demonstrated the efficacy of VNS for the treatment of migraine, anxiety symptoms, depression and epilepsy (54). In this systematic review, VNS together with physical rehabilitation was found to significantly improve upper limb motor function when compared with rehabilitation alone; a similar result to that reported in recently published meta-analyses (50–52). VNS together with physical therapy increases upper limb motor recovery of stroke patients by an average of 7 points in the Fugl-Meyer scale, which could be considered as a clinically significant response (55). In some of the reviewed studies with implanted VNS, a clinically significant response was found in 47 to 88% of patients up to 90 days after the end of in-clinic therapy, supporting potential sustained effects of the intervention on motor recovery after stroke (34, 49).

Results from this meta-analysis suggest that implementation of this intervention at earlier stages of the post-stroke recovery process could have a significantly greater effect in motor rehabilitation. Studies that in included participants where the intervention was, in average, initiated <3 years after the stroke had an estimated increase of eight points in the Fugl-Meyer scale after VNS and motor rehabilitation compared to an estimated increase of five points in the studies that included participants with an average of more than 3 years since the event. Only one study included patients in the sub-acute phase of stroke rehabilitation Wu et al. (43). This study found an average increase of 6.9 points in the Fugl-Meyer scale after 15 days of therapy, which increased to 7.7 points 4 weeks after therapy and was significantly greater than the change observed in the sham group. Given that most of the cortical reorganization processes are expected to occur during the sub-acute phase post stroke (56), this may be the optimal window of recovery to be modulated by the implementation of VNS in combination with physical therapy, however, future clinical studies with larger sample sizes will be necessary to confirm whether earlier administration of this intervention is associated with greater improvement in motor function.

This systematic review identifies several knowledge gaps that should be evaluated in further studies. First, although initial results from studies evaluating transcutaneous VNS are promising, more clinical trials evaluating this technique with larger sample sizes and appropriate control interventions are required to determine a more accurate effect size of this technique in motor recovery after stroke. Other variables that need to be studied include the definition of optimal stimulation parameters and treatment duration, as well as the appropriate timing for the combination of the stimuli with physical rehabilitation protocols. In addition, future studies will need to evaluate whether VNS has differential effects according to the compromised vascular region, severity of the lesion and stroke subtype (e.g., lacunar vs. non-lacunar) among other clinical characteristics that could impact the effectiveness of this intervention.

The systematic review and meta-analysis have some limitations that are important to mention. First, the number of clinical trials, was very low, and one of the included studies had no comparison group. A high statistical heterogeneity between studies was also identified. There are some sources of heterogeneity that could not be evaluated, for example, the day of primary outcome evaluation, physical rehabilitation protocol parameters, the severity of the lesion, and the vascular region affected by the stroke, among others.

We conclude that VNS together with physical rehabilitation improves upper limb motor function in stroke patients. Additionally, VNS is a safe intervention. More studies are needed to evaluate the efficacy and effectiveness of transcutaneous VNS in patients with stroke and to evaluate optimization of its effect according to the timing of the intervention and the use of more effective stimulation parameters.

## Data Availability Statement

The original contributions presented in the study are included in the article/supplementary material, further inquiries can be directed to the corresponding author/s.

## Author Contributions

JR-C independently reviewed the titles and abstracts for an initial assessment of study eligibility criteria. Once the titles and abstracts were reviewed. JR-C and DL-F reviewed the full-text articles to evaluate the inclusion of studies in the analysis. Discrepancies and doubts on the inclusion of articles were resolved by a third investigator RG. JR-C and RG wrote the initial version of the manuscript. JR-C, DL-F, SS-B, and FS-S reviewed the article and made significant contributions to the interpretation of results. All authors contributed to the article and approved the submitted version.

## Funding

This research was conducted with support from the Ministry of Science, Technology and Innovation of Colombia Minciencias (Projects Nos. 656684368671, 656674555082, and 69/2021). Writing style review was supported by Universidad Antonio Nariño.

## Conflict of Interest

The authors declare that the research was conducted in the absence of any commercial or financial relationships that could be construed as a potential conflict of interest.

## Publisher's Note

All claims expressed in this article are solely those of the authors and do not necessarily represent those of their affiliated organizations, or those of the publisher, the editors and the reviewers. Any product that may be evaluated in this article, or claim that may be made by its manufacturer, is not guaranteed or endorsed by the publisher.
